# Relevance of Systematic Pre-Biologic Infectious Screening in Chronic Inflammatory Rheumatic Diseases: A Retrospective Single-Center Study

**DOI:** 10.3390/jcm15124631

**Published:** 2026-06-15

**Authors:** Marie Doussiere, Clémence Jouret, Lara Awad, Pierre-Antoine Bruy, Laetitia Diep, Claire Jesson, Jean-Marc Sobhy-Danial, Franck Grados, Patrice Fardellone, Vincent Goëb

**Affiliations:** 1 Department of Rheumatology, University Hospital of Amiens, 80000 Amiens, France; doussiere.marie@chu-amiens.fr (M.D.); jouret.clemence@chu-amiens.fr (C.J.); awad.lara@chu-amiens.fr (L.A.); bruy.pierre-antoine@chu-amiens.fr (P.-A.B.); diep.laetitia@chu-amiens.fr (L.D.); sobhy-danial.jean-marc@chu-amiens.fr (J.-M.S.-D.); grados.franck@chu-amiens.fr (F.G.); fardellone.patrice@chu-amiens.fr (P.F.); 2 GRAP, INSERM U1247, Université de Picardie Jules Verne, 80000 Amiens, France

**Keywords:** biologic therapy, chronic inflammatory rheumatic diseases, latent tuberculosis, hepatitis B virus, pre-treatment infectious screening

## Abstract

**Background:** Systematic infectious screening is recommended before initiation of biologic therapies in chronic inflammatory rheumatic diseases (CIRDs), yet the clinical impact of this strategy in low-prevalence settings remains insufficiently characterized. This study aimed to evaluate the proportion of abnormal findings and their impact on treatment management. **Methods:** We conducted a retrospective single-center study including adult patients with CIRDs who underwent systematic pre-biologic infectious screening between January 2019 and June 2025. Screening included HIV, hepatitis B virus (HBV), hepatitis C virus (HCV), interferon-γ release assay (IGRA), and chest radiography. The primary outcome was the proportion of abnormal results and their impact on biologic initiation. **Results:** A total of 418 patients was included (mean age 48.2 ± 14.6 years; 69.1% female). No active HIV, HBV, or HCV infections were detected. Past HBV infection markers were identified in 2.6% of patients, and anti-HCV antibodies in 0.7%, all without detectable viremia. None of these findings required modification of biologic therapy. IGRA positivity was observed in 4.3% of patients and indeterminate results were seen in 3.1%. Preventive antituberculous therapy was initiated in most newly identified IGRA-positive cases, leading to delayed biologic initiation in several patients. Chest radiography yielded limited additional diagnostic value. **Conclusions:** In this population, systematic pre-biologic infectious screening identified few clinically actionable viral infections, whereas latent tuberculosis screening represented the main determinant of therapeutic modification. These findings support continued emphasis on tuberculosis risk assessment and warrant further prospective studies to evaluate optimized and potentially targeted screening strategies incorporating cost-effectiveness analyses.

## 1. Introduction

Chronic inflammatory rheumatic diseases, including rheumatoid arthritis, spondyloarthritis, and psoriatic arthritis, are immune-mediated conditions characterized by persistent inflammation, progressive joint damage, and significant functional impairment. Their pathophysiology involves dysregulation of both innate and adaptive immunity, particularly through pro-inflammatory cytokines such as TNF-α, IL-6, and IL-17, which have become key therapeutic targets.

Biologic and targeted synthetic disease-modifying antirheumatic drugs (bDMARDs and tsDMARDs) have substantially improved outcomes in these conditions, whether through cytokine blockade, B-cell depletion, or JAK pathway inhibition. However, their immunomodulatory effects increase susceptibility to opportunistic infections and reactivation of latent infections, particularly tuberculosis and chronic viral hepatitis [1,2]. French national guidelines [3] and the 2022 European Alliance of Associations for Rheumatology (EULAR) recommendations [4] have established systematic pre-biologic infectious screening. These strategies include assessment for latent tuberculosis using interferon-γ release assays combined with clinical evaluation, hepatitis B serology (HBsAg, anti-HBc, anti-HBs), hepatitis C testing with confirmatory RNA analysis, and HIV screening according to risk profile, with appropriate specialist referral and prophylaxis when indicated. Although these strategies aim to minimize infectious complications, their real-world diagnostic yield in low-prevalence European settings remains uncertain. The proportion of abnormal findings that truly impact therapeutic decisions is not well defined, raising questions regarding clinical relevance, cost-effectiveness, and potential treatment delays. Addressing these questions requires evaluating not only the prevalence of abnormal results but also their consequences on therapeutic management, including prophylaxis initiation, and treatment modification or delay. This study therefore aimed to evaluate the clinical utility of systematic pre-biologic infectious screening in patients with chronic inflammatory rheumatic diseases by assessing the prevalence of abnormal results and their impact on treatment management.

## 2. Methods

### 2.1. Study Design and Population

We conducted a retrospective, observational, single-center study in the Department of Rheumatology at Amiens-Picardie University Hospital (France). Adults (≥18 years) with confirmed diagnosis of chronic inflammatory rheumatic disease who underwent pre-biologic infectious screening prior to the initiation of a biologic or targeted synthetic disease-modifying antirheumatic drug between January 2019 and June 2025 were eligible. Pediatric patients were not included, as our rheumatology department exclusively manages adult patients. Patients were excluded if they did not initiate biologic therapy due to the absence of indication, were lost to follow-up after screening, were not followed in the rheumatology department, or had received biologic therapy prior to the study period. The study population was identified through the hospital laboratory database by retrieving all interferon-γ release assays (IGRAs) prescribed by rheumatologists during the study period. After reviewing medical records and application of eligibility criteria, 418 patients were included in the final analysis.

### 2.2. Data Collection

Data were retrospectively extracted from electronic medical records. Collected variables included age at screening, sex, type of chronic inflammatory rheumatic disease (CIRD), and targeted therapy initiated. Pre-biologic infectious screening results were systematically recorded and are summarized in Table 1. Hepatitis B virus (HBV) serological profiles were categorized based on HBsAg, anti-HBc, and anti-HBs results. In cases of anti-HCV positivity, plasma HCV RNA testing was performed to distinguish active infection (detectable RNA) from past infection (undetectable RNA). Tuberculosis screening was performed using an interferon-γ release assay (IGRA; QuantiFERON-TB Gold). In cases of indeterminate results, repeat testing was performed, and if indeterminate results persisted, an alternative IGRA (T-SPOT^®^.TB) was carried or the IGRA was repeated after an interval, according to individual clinician practice. Chest radiography findings were categorized according to radiological interpretation, including abnormalities suggestive of active or previous tuberculosis. Delays in biologic initiation attributable to screening findings were recorded when applicable.

### 2.3. Cost Analysis

The theoretical cost of pre-biologic infectious screening was estimated based on national reimbursement rates established by the French *Nomenclature des Actes de Biologie Médicale* (NABM) and the *Classification Commune des Actes Médicaux* (CCAM). Unit costs were applied to each screening component for all included patients to calculate the total and mean per-patient screening costs. The cost per abnormal finding was derived by dividing the total screening cost by the number of clinically relevant abnormalities identified. Unit costs were €12 for HIV serology, €40 for HBV serology, €13 for HCV serology, €40 for IGRA testing, and €32 for chest radiography.

### 2.4. Outcomes and Statistical Analysis

The primary outcome was the proportion of abnormal findings identified during pre-biologic infectious screening and their impact on treatment initiation. A descriptive analysis was performed. Categorical variables are presented as numbers and percentages, and continuous variables as mean ± standard deviation.

### 2.5. Ethical Approval

The study was approved by the Clinical Research and Innovation Department of Amiens-Picardie University Hospital (Project No. PI2025_843_0205). In accordance with French regulations for retrospective non-interventional studies, informed consent was not required.

## 3. Results

### 3.1. Baseline Characteristics

A total of 418 patients were included in the analysis. Baseline characteristics are summarized in Table 2. The mean age at the time of pre-biologic screening was 48.2 ± 14.6 years, and 289 patients (69.1%) were female. Twelve patients (2.9%) were born outside France, including four (0.96%) from tuberculosis-endemic regions. Impaired oral health status was documented in ten patients (2.4%). Prolonged corticosteroid therapy (≥3 months) prior to biologic initiation was recorded in 123 patients (29.4%). Eighty-five patients (20.3%) had previously received a biologic agent, which had been discontinued before the current therapeutic strategy. Six patients (1.4%) reported drug use, including four with intravenous use; four patients were receiving chemotherapy, and two had uncontrolled diabetes. No cases of primary immunodeficiency, chronic kidney disease, organ transplantation, advanced malignancy, or cirrhosis were documented. The most frequent diagnoses were axial spondyloarthritis (44.7%), rheumatoid arthritis (33.3%), and psoriatic arthritis (16.8%). An anti-tumor necrosis factor (anti-TNF) agent was prescribed in 80.9% of patients, followed by interleukin inhibitors (8.1%), B-cell-targeted therapies (7.2%), and Janus kinase inhibitors (3.8%). Before biologic initiation, 62.2% of patients had received at least one conventional synthetic DMARD, most commonly methotrexate. The mean duration between symptom onset and pre-biologic screening was 8.2 ± 9.3 years. The mean interval between screening and biologic initiation was 0.28 ± 0.75 years and was less than one year in 76.6% of cases. Biologic initiation was delayed in 39 patients (9.3%), most frequently due to dental care (*n* = 17) or initiation of latent tuberculosis treatment (n = 15).

### 3.2. Results of Pre-Biologic Infectious Screening

**Figure 1:**
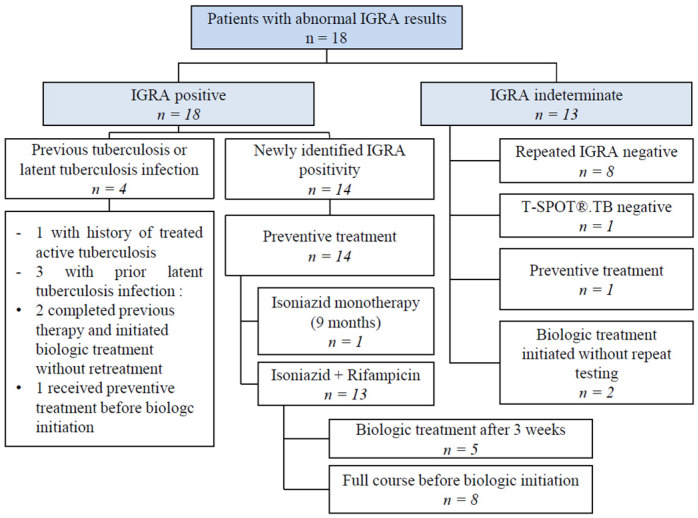
Clinical management of patients with positive or indeterminate IGRA results during pre-biologic infectious screening.

No patient had a positive HIV serology. HBV screening showed that 50.0% of patients had a completely negative profile, 46.2% had a vaccinated profile, and 2.6% had markers consistent with past infection. No cases of chronic HBV infection were identified. Anti-HCV antibodies were detected in three patients (0.7%), all with undetectable HCV RNA. No active HCV infection was observed. Detailed viral screening results are presented in Table 3. IGRA results were negative in 92.6% of patients, positive in 4.3%, and indeterminate in 3.1%. All patients with positive IGRA results had normal chest radiographs. The management and outcomes of patients according to IGRA results are detailed in Figure 1. Among the 18 patients with positive IGRA results, one had a history of treated active tuberculosis and three had prior latent tuberculosis infection. Of the latter, two had completed previous therapy and initiated biologic treatment without retreatment, while one received preventive therapy before biologic initiation. The remaining 14 patients had newly identified IGRA positivity; 13 received preventive therapy with isoniazid plus rifampicin (eight completed the full course before biologic initiation and five initiated biologic therapy after three weeks of prophylaxis), and one received nine months of isoniazid monotherapy. Among the 13 patients with indeterminate IGRA results, eight had negative repeat testing, one had a negative T-SPOT^®^.TB assay, one received preventive therapy, and two initiated biologic treatment without repeat testing. One patient had a prior history of treated latent tuberculosis infection. Notably, three patients with previously treated latent tuberculosis and four originating from endemic regions had negative IGRA results. Chest radiography was abnormal in three patients (0.7%), with previously known nodular lesions in two cases. All underwent thoracic computed tomography, and biologic therapy was initiated without additional restrictions.

### 3.3. Screening Costs

For the 418 patients who underwent complete screening, the total theoretical cost was approximately €53,504, corresponding to a mean cost of €128 per patient. A total of 53 biological or radiological abnormalities were identified. When considering the overall screening cost relative to the number of abnormalities detected, the estimated cost per abnormal finding was approximately €1010.

## 4. Discussion

In this retrospective single-center study including 418 patients, systematic pre-biologic infectious screening identified a limited number of clinically actionable abnormalities. No active HIV, HBV, or HCV infections were detected, and the overall impact on therapeutic management was primarily driven by tuberculosis screening findings. These results largely confirm the validity of current recommendations in a low-prevalence European setting, while raising questions about the optimization of screening strategies in such contexts. However, the retrospective single-center design and the relatively low prevalence of infectious risk factors in our cohort limit the generalizability of these findings.

**HIV screening**. The absence of patients with HIV infections aligns with European rheumatology data reporting very low HIV seroprevalence, including the 0.12% prevalence observed in the French ESPOIR cohort of patients with early rheumatoid arthritis undergoing systematic screening [5].

**HBV screening and vaccination.** Regarding HBV infection, our findings agree with real-world rheumatology data indicating that chronic active infection is uncommon, whereas markers of past infection are more frequently encountered. Ditto et al. (2021) [6] reported an overall HBV prevalence of 15.7% in a rheumatologic population, including 0.4% chronic infection and 12.6% resolved infection. Notably, 12 patients (6.2%) with evidence of HBV infection experienced viral reactivation, all with serological profiles consistent with past infection (two patients HBsAg-negative, anti-HBc-positive, anti-HBs-negative; 10 patients HBsAg-negative, anti-HBc-positive, anti-HBs-positive), demonstrating that reactivation is not restricted to patients who are HBsAg-positive. One case occurred despite lamivudine prophylaxis. These observations are consistent with the hepatology literature indicating that although reactivation risk is highest in patients who are HBsAg-positive, it remains clinically relevant in individuals who are HBsAg-negative and anti-HBc-positive, particularly under potent immunosuppression such as B-cell depletion [7,8]. The 2022 EULAR and 2013 French recommendations therefore advocate for systematic screening using HBsAg, anti-HBc, and anti-HBs prior to biologic therapy, with antiviral prophylaxis or monitoring tailored to serological status and treatment type [3,4]. Vaccination is recommended for patients who are fully seronegative. In our cohort, 46.2% of patients had a vaccinated profile. Historical European data reported considerably lower vaccination coverage, such as 7.8% in the cohort described by Feuchtenberger et al. (2016) [9], while more recent data in immune-mediated diseases continue to suggest suboptimal coverage (approximately 21% in a 2025 study) [10]. These findings suggest relatively satisfactory vaccination coverage in our population while underscoring the importance of systematic pre-biologic immunization strategies.

**HCV screening.** Concerning HCV infection, our results are comparable to real-world European data. In a German cohort of 697 patients, Feuchtenberger et al. (2025) [11] reported anti-HCV positivity in 0.4% of cases. In contrast, higher seroprevalence has been described in different epidemiological contexts, such as the Pakistani cohort reported by Qamar et al. (2025) [12], in which 9.6% of patients tested positive for anti-HCV antibodies but none had detectable RNA after confirmation. Regarding safety under biologic therapy, Brunasso et al. (2011) [13] identified only one confirmed case of hepatitis C worsening among 110 anti-TNF-treated patients, and Snast et al. (2017) [14] reported reactivation events in three of 97 patients (annual incidence of 2.42%). Collectively, these data indicate that biologic therapy can generally be administered safely in patients without detectable viremia, provided there is appropriate monitoring and hepatology collaboration when necessary. This approach is consistent with EULAR 2022 and French 2013 recommendations, which emphasize the distinction between active (RNA-positive) and past infection prior to biologic initiation [3,4].

**Tuberculosis screening.** Tuberculosis screening represented the most clinically impactful component of pre-biologic evaluation. IGRA positivity (4.3%) is within the 4–13% range reported in European cohorts, depending on epidemiological context and screening strategy. Kleinert et al. [15] reported a TST positivity of 11.3% among over 1500 patients eligible for biologic therapy, with estimated latent tuberculosis prevalence ranging from 7.9% to 11.1% depending on the screening approach used. Hsia et al. [16] observed at least one positive screening test in 13.8% of patients in golimumab trials, with low concordance between TST and the IGRA. Conversely, de Jong et al. [17] reported 4% IGRA positivity in a Dutch low-endemic cohort of 549 patients, while Feuchtenberger et al. (2025) [11] reported a prevalence of 14.2% in Germany despite low endemicity. Our data further illustrate the imperfect sensitivity of IGRA testing: three patients with previously treated latent tuberculosis and four originating from endemic regions had negative IGRA results. Indeterminate results occurred in 3.1% of cases. These findings reflect the known limitations of immune-based assays, particularly in immunosuppressed populations. The GETAID cohort described by Abitbol et al. [18] demonstrated that negative baseline screening does not fully eliminate tuberculosis risk under anti-TNF therapy, with 44 cases developing tuberculosis despite negative initial screening. Chest radiography had limited incremental value in our asymptomatic cohort, with only three abnormalities detected, all previously known. Several studies suggest minimal additional diagnostic yield of routine imaging in low-risk, IGRA-negative patients [19,20], and recent consensus statements acknowledge that imaging may be omitted in carefully selected low-risk individuals, although this remains dependent on national algorithms and local epidemiology [4,20,21]. In cases of newly identified latent tuberculosis, a key clinical question concerns the potential delay in biologic initiation imposed by prophylactic treatment. According to current French and EULAR recommendations, treatment consists of isoniazid combined with rifampicin for three months, and biologic therapy may be initiated as early as three weeks after the start of prophylaxis, provided there is good tolerability. This relatively short delay limits the clinical impact on disease management in most patients.

**Risk profile.** In our cohort, a substantial proportion of patients presented no identifiable infectious risk factors at the time of screening, including no history of exposure, no endemic region origin, and no immunocompromising comorbidities. Whether some screening components could be selectively omitted in such patients without compromising safety remains an open question. Current guidelines do not formally endorse a risk-stratified approach, and the low but non-negligible rates of reactivation observed even in apparently low-risk patients argue for caution. Nevertheless, our findings support further evaluation of targeted screening strategies in prospective studies.

**Health economic considerations.** The cost analysis presented in this study is purely descriptive and should not be interpreted as a formal cost-effectiveness analysis. No economic modeling, comparison with alternative screening strategies, or downstream complication analysis were performed. Nevertheless, the estimated cost per abnormal finding of approximately €1010 raises questions regarding the economic efficiency of fully systematic screening in low-prevalence settings.

**Initiation treatment delays** Our study did not collect data on the actual delays in biologic initiation attributable to screening findings. The minimum three-week delay imposed by latent tuberculosis prophylaxis before biologic initiation is generally acceptable in clinical practice. In rheumatoid arthritis, where structural joint damage represents the main concern, most patients are already receiving methotrexate at the time of biologic initiation, which provides adequate disease control during this short interval except in cases of contraindication. Beyond infectious risk, forgoing systematic screening in low-risk patients could also reduce the number of consultations required before biologic initiation, a consideration given the limited access to specialists in many healthcare systems. However, as illustrated in our cohort, abnormal findings can occur even in apparently low-risk patients, arguing against a blanket abandonment of pre-therapeutic screening.

Overall, our findings support the continued need for pre-therapeutic screening, while advocating for a rational adaptation of its components based on local epidemiology and individual patient risk profiles. Prospective multicenter studies, incorporating detailed medico-economic analyses and stratification of patients according to their infectious risk factors, are needed to determine whether a targeted approach to pre-therapeutic screening could optimize patient management, reduce delays in initiating targeted therapies, and generate healthcare cost savings, while maintaining an optimal level of patient safety.

## Figures and Tables

**Table 1:** Classification of pre-biologic infectious screening results.

Variable	Categories
HIV serology	Negative/Positive
HBV serological profile	Negative (HBsAg−, anti-HBc−, anti-HBs−) Vaccinated (HBsAg−, anti-HBc−, anti-HBs+) Past infection (HBsAg−, anti-HBc+ with or without anti-HBs) Chronic infection (HBsAg+)
HCV screening	Negative (anti-HCV−) Past infection (anti-HCV+, RNA−) Active infection (anti-HCV+, RNA+)
IGRA result	Negative/Positive/Indeterminate
Chest X-ray	Normal/Abnormal

**Table 2:** Demographic and clinical characteristics of the study population.

Patient Population (n = 418)	n (%)
Mean age ± SD	48.16 ± 14.59
Female	289 (69.14%)
MSM	3 (0.72%)
Foreign-born	12 (2.87%)
Poor housing conditions	1 (0.24%)
Poor oral health	10 (2.40%)
Immunosuppression	
Corticosteroid therapy	123 (29.43%)
Prior biologic therapy	85 (20.33%)
Other	106 (23.35%)
Chemotherapy	4 (3.78%)
Uncontrolled diabetes	2 (1.89%)
Substance use	6 (5.66%)
Multiple risk factors	8 (7.55%)
Tuberculosis risk	
History of latent tuberculosis infection	8 (1.91%)
Origin from high tuberculosis-endemic area	4 (0.96%)

SD: standard deviation. MSM: men who have sex with men.

**Table 3:** Results of pre-biologic infectious screening.

HIV	n (%)
Positive	0
**HBV**	
Negative	209 (50%)
Vaccinated	193 (46.17%)
Borderline immunity	5 (1.20%)
Resolved infection	11 (2.63%)
Active infection	0
**HCV**	
Negative	415 (99.28%)
Resolved infection	3 (0.72%)
Active infection	0
**IGRA**	
Negative	387 (92.59%)
Positive	18 (4.3%)
Indeterminate	13 (3.11%)
**Chest radiograph**	
Normal	415 (99.28%)

## Data Availability

The data presented in this study are available on request from the corresponding author.

## References

[1] Fragoulis G.E., Sipsas N.V. (2019). When rheumatology and infectious disease come together. Ther. Adv. Musculoskelet. Dis..

[2] Hsu C.-Y., Ko C.-H., Wang J.-L., Hsu T.-C., Lin C.-Y. (2019). Comparing the burdens of opportunistic infections among patients with systemic rheumatic diseases: A nationally representative cohort study. Arthritis Res. Ther..

[3] Goëb V., Ardizzone M., Arnaud L., Avouac J., Baillet A., Belot A., Bouvard B., Coquerelle P., Dadoun S., Diguet A. (2013). Recommendations for using TNFα antagonists and French Clinical Practice Guidelines endorsed by the French National Authority for Health. Jt. Bone Spine.

[4] Fragoulis G.E., Nikiphorou E., Dey M., Zhao S.S., Courvoisier D.S., Arnaud L., Atzeni F., Behrens G.M., Bijlsma J.W., Böhm P. (2023). 2022 EULAR recommendations for screening and prophylaxis of chronic and opportunistic infections in adults with autoimmune inflammatory rheumatic diseases. Ann. Rheum. Dis..

[5] Varache S., Narbonne V., Jousse-Joulin S., Guennoc X., Dougados M., Daurès J.P., Devauchelle-Pensec V., Saraux A. (2011). Is routine viral screening useful in patients with recent-onset polyarthritis of a duration of at least 6 weeks? Results from a nationwide longitudinal prospective cohort study. Arthritis Care Res..

[6] Ditto M.C., Parisi S., Varisco V., Talotta R., Batticciotto A., Antivalle M., Gerardi M.C., Agosti M., Borrelli R., Fusaro E. (2021). Prevalence of hepatitis B virus infection and risk of reactivation in rheumatic population undergoing biological therapy. Clin. Exp. Rheumatol..

[7] Terrault N.A., Lok A.S.F., McMahon B.J., Chang K.M., Hwang J.P., Jonas M.M., Brown R.S., Bzowej N.H., Wong J.B. (2018). Update on prevention, diagnosis, and treatment of chronic hepatitis B: AASLD 2018 hepatitis B guidance. Hepatology.

[8] Myint A., Tong M.J., Beaven S.W. (2020). Reactivation of Hepatitis B Virus: A Review of Clinical Guidelines. Clin. Liver Dis..

[9] Feuchtenberger M., Schäfer A., Philipp Nigg A., Rupert Kraus M. (2016). Hepatitis B Serology in Patients with Rheumatic Diseases. Open Rheumatol. J..

[10] Bellofatto I.A., Paci V., Conti F., Santoboni G., Sebastiani G.D., Cattaruzza M.S., Mazzanti C., Salemi S., Sesti G., Tesoriere E. (2025). Coverage and Drivers of Vaccinations in Patients with Autoimmune Rheumatic Diseases: An Italian Multicentric Study. Vaccines.

[11] Feuchtenberger M., Kovacs M.S., Nigg A., Schäfer A. (2025). Detection of substantial numbers of latent tuberculosis and positive hepatitis B serology results in rheumatology patients preparing to receive intensified immunosuppressive therapy in a low-prevalence country: Why screening still matters. Clin. Rheumatol..

[12] Qamar H.Y., Saeed M.A., Hameed M.R., Aamer M., Arshad U., Lal A. (2025). Clinical Audit of screening Latent TB, Hepatitis-C, and Occult Hepatitis-B in Rheumatoid arthritis’ patients starting biologic or targeted synthetic DMARDS. Pak. J. Med. Sci..

[13] Brunasso A.M., Puntoni M., Gulia A., Massone C. (2011). Safety of anti-tumour necrosis factor agents in patients with chronic hepatitis C infection: A systematic review. Rheumatology.

[14] Snast I., Atzmony L., Braun M., Hodak E., Pavlovsky L. (2017). Risk for hepatitis B and C virus reactivation in patients with psoriasis on biologic therapies: A retrospective cohort study and systematic review of the literature. J. Am. Acad. Dermatol..

[15] Kleinert S., Tony H.P., Krueger K., Detert J., Mielke F., Rockwitz K., Schwenke R., Burmester G.R., Diel R., Feuchtenberger M. (2012). Screening for latent tuberculosis infection: Performance of tuberculin skin test and interferon-γ release assays under real-life conditions. Ann. Rheum. Dis..

[16] Hsia E.C., Schluger N., Cush J.J., Chaisson R.E., Matteson E.L., Xu S., Beutler A., Doyle M.K., Hsu B., Rahman M.U. (2012). Interferon-γ release assay versus tuberculin skin test prior to treatment with golimumab, a human anti-tumor necrosis factor antibody, in patients with rheumatoid arthritis, psoriatic arthritis, or ankylosing spondylitis. Arthritis Rheum..

[17] De Jong M.J., Roosen D., Van Tubergen A. (2018). The Prevalence of Latent Tuberculosis and Hepatitis B After Systematic Screening of Patients Prescribed Biological Therapy in a Low-endemic Area. J. Crohns Colitis.

[18] Abitbol Y., Laharie D., Cosnes J., Allez M., Nancey S., Amiot A., Aubourg A., Fumery M., Altwegg R., Michetti P. (2016). Negative Screening Does Not Rule Out the Risk of Tuberculosis in Patients with Inflammatory Bowel Disease Undergoing Anti-TNF Treatment: A Descriptive Study on the GETAID Cohort. J. Crohns Colitis.

[19] Bonde Christiansen S., Ainsworth M.A. (2024). The role of chest X-rays when screening for latent tuberculosis infection in patients with inflammatory bowel disease before starting biologic treatment. Scand. J. Gastroenterol..

[20] Tan D.Y.L., Kim Y.H., Darmalingam M., Mohandas P. (2025). Are chest X-rays still necessary for prebiologic tuberculosis screening? A retrospective audit. Clin. Exp. Dermatol..

[21] Rath E., Bonelli M., Duftner C., Gruber J., Mandl P., Moazedi-Furst F., Pieringer H., Puchner R., Flick H., Salzer H.J.F. (2022). National consensus statement by the Austrian Societies for Rheumatology, Pulmonology, Infectiology, Dermatology and Gastroenterology regarding the management of latent tuberculosis and the associated utilization of biologic and targeted synthetic disease modifying antirheumatic drugs (DMARDs). Wien. Klin. Wochenschr..

